# The immunomodulatory role of IDO1-Kynurenine-NAD^+^ pathway in switching cold tumor microenvironment in PDAC

**DOI:** 10.3389/fonc.2023.1142838

**Published:** 2023-06-30

**Authors:** R. I. Anu, Kai-Keen Shiu, Khurum Hayat Khan

**Affiliations:** ^1^ Department of Cancer Biology and Therapeutics, Precision Oncology and Multi-Omics Clinic, Genetic Counseling Clinic, Department of Clinical Biochemistry, MVR Cancer Centre and Research Institute, Calicut, Kerala, India; ^2^ Gastrointestinal Oncology Service, University College London Hospitals National Health Services (NHS) Foundation Trust, London, United Kingdom; ^3^ Universtiy College London (UCL) Cancer Institute, University College London Hospitals National Health Services (NHS) Foundation Trust, London, United Kingdom; ^4^ Whittington Health, National Health Services (NHS), London, United Kingdom

**Keywords:** pancreatic cancer, metabolome, immune microenvironment, Indolamine 2,3-dioxygenase 1, Kynurenine, NAD, epacadostat, NAMPT inhibitor

## Abstract

Pancreatic ductal adenocarcinoma (PDAC) is the most common exocrine tumor of the pancreas characterized by late diagnosis, adverse overall 5-year survival, a higher propensity for metastatic disease, and lack of efficacy of systemic therapy options. These adverse outcomes can be partly attributed to complex tumor microenvironment (TME). Over the past decade, immunotherapy has revolutionized the management of certain cancers; thus far, the immunologically ‘non-inflamed’ tumor microenvironment in PDACs has proven to be challenging. Indolamine 2,3-dioxygenase 1 (IDO1) is the rate-limiting enzyme in the catabolic pathway of L-Tryptophan, an essential amino acid, that gives rise to the immunosuppressive metabolite Kynurenine. IDO1, Indolamine 2,3-dioxygenase 2 (IDO2), and Tryptophan 2,3-dioxygenase (TDO) are the key enzymes in the tryptophan catabolic pathway but we focus on the role of the predominant enzyme form IDO1 in this review. Nicotinamide phosphoribosyl transferase (iNAMPT) regulates the intracellular concentration of NAD and is upregulated in the tumor. In light of the potential role of IDO1 as a driver of hostile TME in PDAC and NAD^+^ as a key coenzyme in anti-tumor immune response, this review urges focus on extensive research and initiation of clinical trials using IDO1 and NAMPT inhibitors in pancreatic cancer in the future.

## Introduction

Pancreatic tumors, specifically pancreatic ductal adenocarcinoma (PDAC) composing 90% of the exocrine tumors of the pancreas are largely known for diagnosis at later stages, poor overall 5-year survival, a higher propensity for metastatic disease, and poor response to immunotherapy. A multitude of factors is responsible for the grim profile of the disease. As research is underway to uncover the molecular blueprint of PDAC and its unique tumor microenvironment (TME), we intend to review the significant, yet underappreciated intracellular metabolic contribution of tumor cells and TME in rendering PDAC an immune-privileged malignancy (1).

### The immune microenvironment of Pancreatic cancer

The tumor microenvironment (TME) influences the malignant process of pancreatic ductal adenocarcinoma (PDAC) more than any other cancer type. Pancreatic TME (2) constitutes a complex interaction between tumor cells, stromal cells such as cancer-associated fibroblasts (CAFs), endothelial cells, perivascular cells, immune cells such as tumor-infiltrating lymphocytes (TILs), tumor-associated macrophages (TAMs), and extracellular matrix elements such as collagen (3, 4), hyaluronic acid (5, 6), fibronectin (7–9), laminin (10–14), and sulfated glycosaminoglycans (15) in an acidic pH (16), resulting in a heavy stromal desmoplastic reaction. In terms of multi-omic approaches, genomic and transcriptomic advances have unearthed the molecular biology of pancreatic cancer to an extent. However, the role of immunotherapy is yet a challenging subject in PDAC. To help uncover newer biomarkers and address the unmet need to comprehend the immunologically ‘non-inflamed’ tumor microenvironment, we require a better understanding of the tumor proteome and metabolome, and the crosstalk between T cells and intermediary molecules that are crucial in signaling pathways and gene regulation (17). The metabolome is the unexcavated effector that executes immune-mediated signals within a malignant cell. From that perspective, a unique ketone Kynurenine has stood out owing to its novel immunoregulatory properties. Indolamine 2,3-dioxygenase 1 (IDO1) is a rate-limiting enzyme in the catabolic pathway of L-tryptophan, an essential amino acid, that gives rise to the immunosuppressive metabolite L-kynurenine. IDO1, Indolamine 2,3-dioxygenase 2 (IDO2), and Tryptophan 2,3-dioxygenase (TDO) are the key enzymes in the pathway, but we focus on the predominant enzyme form IDO1 in this review. The role of Kynurenine in immune privilege and its blood levels in cancer has been studied in cancers of the colon, stomach, breast, and prostate (18). However, its role in the immune microenvironment of Pancreatic cancer is yet to be investigated in-depth. Here we intend to focus solely on the function and duality of immune cells within the pancreatic ‘cold’ TME from the perspective of tumor-metabolomic crosstalk.

## The lymphoid and myeloid compartments hold the recipe for an *indifferent* TME

Pancreatic TME houses a heterogeneous population of immune cells skewed to immune-suppressive functionality than with tumor-inhibiting characteristics. This is initially driven by the unique genomic alterations observed in PDAC.

### The interplay of immune cell regulation, TME behavior, and cellular energetics

A harsh TME studded with immunosuppressive or pro-tumor myeloid-derived suppressor cells (MDSCs) and tumor-associated macrophages (TAMs) drives the PDAC cells into extensive metabolic rewiring (19) involving central carbon metabolism, glucose, and glutamine utilization, but generating lesser ATP. This rewiring is predominantly driven by oncogenic *KRAS* variants. Eventually, the tumor cells and T-cells in the TME compete for glucose as activation of T-cells requires upregulation of GLUT-1 glucose transporter *via* TCR and Akt activation. As the supply of glucose is diminished along with glutamine and arginine, the T-cells lose functional capacity (20). With an active IDO-1 enzyme breaking down tryptophan to release immunosuppressive metabolites, the immune cells face an added disadvantage. Furthermore, the IDO1- Kynurenine pathway is a *de novo* source of nicotinamide dinucleotide (NAD) which is metabolized to adenosine which in turn binds to T cell adenosine receptor A2R that inhibits effector T cells and stimulates Tregs. Hypoxic TMEs are under higher influence of this adenosinergic axis activation and immunosuppression. The immunosuppressive-hypoxic environment is further stiffened by intracellular nicotinamide phosphoribosyl transferase (iNAMPT), a crucial enzyme in NAD biosynthesis, *via* the NAD/SIRT1/HIF-1α axis acts on the mobilization of MDSCs by inhibiting *CXCR4* transcription (21). The energetics within the cells define tumor immune escape, the potential for invasiveness, and metastasis.

Increased levels of kynurenine prevent the proliferation of NK cells and T cells by interactions with the aryl hydrocarbon (AhR) receptor. The general control non-deprepressible-2 (GCN2) and mammalian target of rapamycin (mTOR) kinases are also believed to be involved in this effect. Kynurenine *via* the AhR and FoxP3 transcription factor also urges the differentiation of naïve CD4^+^ T-cells to T- regulatory cells that are immunosuppressive by nature (22). Tryptophan metabolism birthing kynurenine is a pathway that is a proven generator of one-carbon units for the pancreatic stellate cells (PSCs), a precursor of CAFs to help maintain tumor growth by purine nucleotide synthesis (23). It is unknown if the primary aim of the pathway is providing immune privilege to tissues or generating one-carbon units and maintaining redox balance in the tissue.

There is a notable differential regulation of T_H_1/T_H_2 by IDO. Stimulation of IDO activity by positive signals or lack of inhibitory molecules such as DNAX-activation protein 12 (DAP12) appears to decrease T_H_1 cellular responses. Further, 3-hydroxyanthranilic (3-HAA) and quinolinic acid metabolites of kynurenine have been found to induce selective apoptosis of murine T_H_1 cells but not of T_H_2 cells. It could be a specific negative feedback mechanism for T_H_1 cells (24). Yet, induction of apoptosis of macrophages required >10-fold concentrations of 3-HAA in a study by Fallarino F et al. In addition, this apoptosis is surprisingly not mediated by Fas/Fas ligand and cytochrome c (25). T_H_17 cells (CD4^+^IL-17^+^) are effector T cells found in the pancreatic TME. Along with IL-17A, they are involved in immune regulation. However, He S et al. remarks “the mechanism for regulating the balance of T_H_17/Treg cells in the tumor microenvironment needs to be further elucidated” (26). We now recognize that anthranilic acid (AA) and 3-hydroxyanthranilic acid (3-HAA) can abolish the function of T_H_17 cells in a dose-dependent manner (27). The anti-tumor function of T_FH_ cells (Follicular helper T cells) in PDAC is gridlocked by the PD-L1/PD-1 signaling pathway (28). B cells have been implicated in immune tolerance, but the exact mechanism is still under investigation. Regulatory B cells (iBregs) have been found to suppress immune responses *via* IL-10 which is the cytokine responsible for converting naïve CD4^+^ T cells to CD4^+^ CD25^+^ Foxp3^+^ Tregs that produced TGF-β. Bregs have also been found to cause T cell apoptosis by cell-to-cell physical contact. Moreover, a CTLA-4 dependent TGFβ/IDO axis in B cells can induce IDO1 and convert them to induced iBregs that could create Tregs, Type 1 T-regulatory cell (Tr1), and T_H_3 cells which in turn suppress T_H_1 cell induction (29, 30).

**Figure 1:**
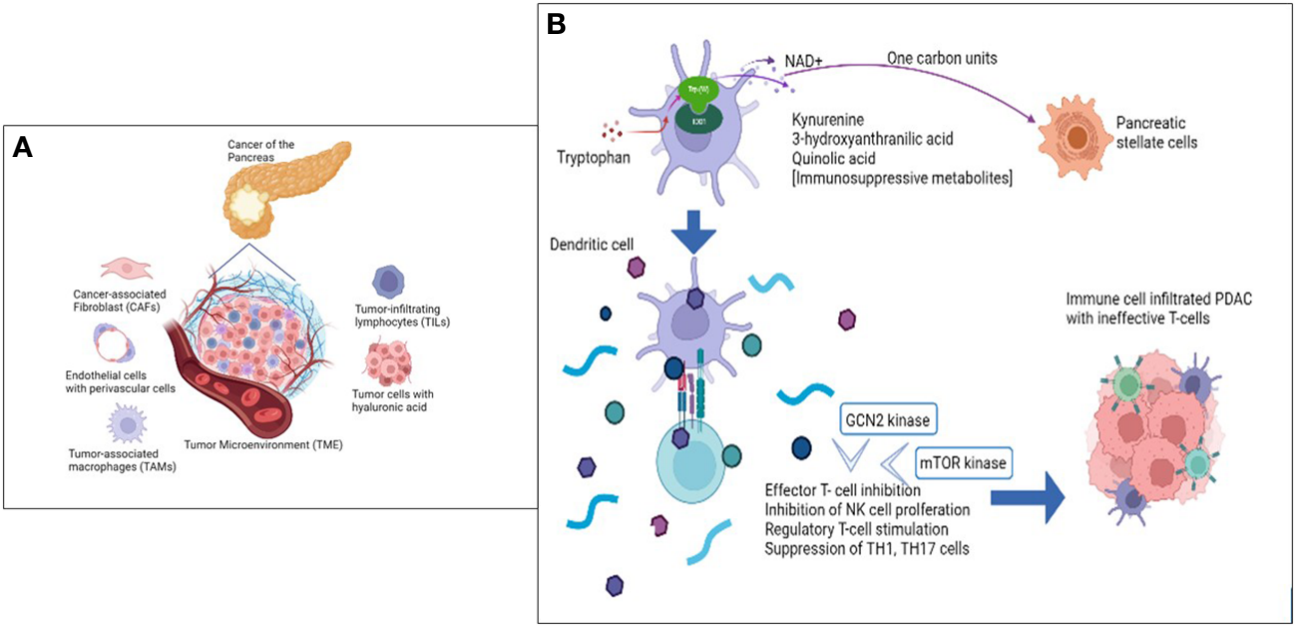
The Tumor microenvironment metabolome is key in PDAC immune modulation. **(A)** Cellular components of the Tumor Microenvironment (TME) that primarily influence and contribute to ‘non-inflamed’ tumor architecture. **(B)** Role of Tryptophan and IDO1 enzyme in immune inhibition of PDAC targeting Immune cell repertoire.

Kynurenine pathway gene expression and immune cell inhibitory checkpoints (T cell signatures) are inversely correlated. Higher Kynurenine and kynurenic acid levels were also found to cause anergy phenotype and CD4^+^ T-cell exhaustion (**Figures 1A, B**). Fundamentally, kynurenine has an unmistakable role in cancer immune escape, making IDO1 a potential candidate to assist immunotherapy (31).

## A quest beyond cellular elements to the realm of the metabolome

### Tumor cell machinery utilizing Kynurenine for Immune suppression in the pancreas

In the normal state, mature dendritic cells in lymphoid organs, the vagina, and placental and lung endothelial cells express the enzyme IDO (32). The cellular localization of the enzyme is cytosol (33). Evolutionarily, induction of IDO and generation of kynurenine metabolites have been proposed to be for two reasons: a genome-immune protective mechanism during the phagocyte-induced respiratory burst that generates reactive oxygen species (ROS) including superoxide anion. This event induces the production of IDO enzyme that uses the superoxide anion to break the pyrrole ring of tryptophan, but few anions escape to enter the nucleus. To thwart pathogen invasion, ROS generation inadvertently causes DNA strand damage, thus inducing PARP molecules that are necessary for DNA damage repair (DDR). This process drains the NAD^+^ pool which is in turn replenished by the IDO-Kynurenine-NAD^+^ pathway (34). The second evolutionary advantage is that Kynurenine and its metabolites play a significant role in immune privilege. IDO bridges the innate and adaptive immune systems. The adenosine/purinergic pathway, cytotoxic T lymphocyte antigen-4 (CTLA-4) and programmed cell death-1 (PD-L1) aid Kynurenine and metabolites to induce immune privilege in certain sites. This is attained by a) tryptophan exhaustion *via* induction of GCN2 and suppression of mTOR1 pathways that lead to T cell inhibition, b) induction of T_H_17 cells and transdifferentiation by dendritic cells and macrophages which are induced by the effect of Kynurenine on aryl hydrocarbon receptor (AhR), c) using PTEN protein to aid in the differentiation of CD4 T cells into Treg cells, d) inhibition of IL-2 that impeded CD4 T cell survival (35). However, the intended metabolic effect is sabotaged when the scenario changes from a healthy state to cancer.

**Figure 2:**
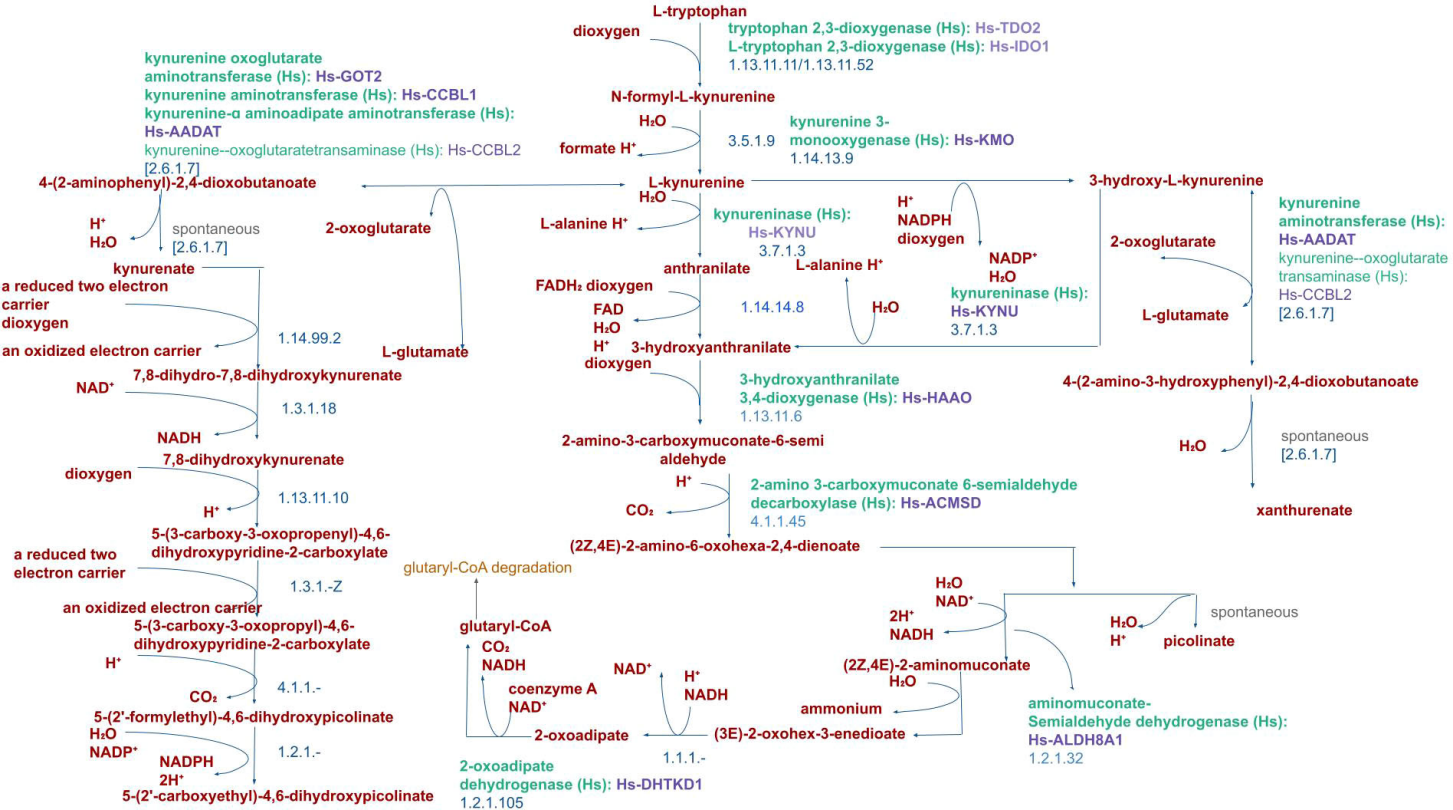
Pathway of Tryptophan catabolism in Humans. Catabolism of L-tryptophan by the human IDO1 enzyme produces L-kynurenine and immunosuppressive metabolites such as 3-hydroxyanthranilic acid (3-HAA) and quinolinic acid metabolites.

In KPC mice bearing PDAC, restriction of serine has been found to be of no effect. This is postulated to be due to a) increased synthesis of serine *de novo*, or b) due to tryptophan catabolism offering one-carbon units. The latter is supported by the finding that interstitial fluid analysis shows severe tryptophan depletion (36, 37). Immune cells (T cells and macrophages) are sources of the cytokine IFN-γ (38) which is the sole inducer of the IDO enzyme. As immune cell infiltration in PDAC increases (39), it also increases tryptophan metabolism. In the catabolic pathway, L-tryptophan is initially converted to N-formyl-l-kynurenine by the rate-limiting enzymes. This metabolite is further converted to L-kynurenine which undergoes further downstream catabolism to form xanthurenate, glutaryl-CoA, and picolinate apart from generation of NAD^+^. Immunomodulation of the TME is however brought about by the intermediary metabolites and compounds formed during Tryptophan catabolism, as elaborated early in the review (**Figure 2**) (40). Whether the immunosuppressive milieu that follows this pathway activation is the intended effect or the bystander effect, is a crucial and intriguing argument that we are yet to unravel.

### Hypoxia and Kynurenine-dependent immune modulation conundrum in PDAC

Hypoxia as an independent factor of tumor aggressiveness is pronounced in Pancreatic cancer when compared to other tumor types. An elevated hypoxia-inducible factor-1 (HIF-1) is an ominous marker for poor prognosis and metastatic events due to the activation of epithelial to mesenchymal transition (EMT). The extensive desmoplastic reaction seen in pancreatic tumor tissue is also directly related to hypoxia (41). Bao B et al. (42) have studied pancreatic tumor cells to uncover molecular mechanisms of hypoxia and discovered that hypoxia-induced increased levels of VEGF, IL-6, and gene expression of cancer stem cell signature genes *Nanog, Oct4*, and *EZH2* that are implicated in tumor invasiveness and aggressive biology. In addition, the pancreatospheres showed increased expression of *miR-21* and *miR-210.* Vieira NF et al. (43) have shown that tissue expression of the two micro-RNAs combined with CA 19-9 had a diagnostic accuracy of 100% in PDAC. The introduction of an inhibitory molecule, a curcumin-derived novel analogue (CDF) revealed a reversal of the hypoxia-induced molecular signature in the tumor cells (42). Intracellular and tumoral hypoxia instinctively shifts the cellular metabolism from oxidative phosphorylation (OXPHOS) yielding high ATP to anaerobic glycolysis with the formation of lactate, bypass of the tricarboxylic acid cycle, and loss of ATP. Genomic factors such as *KRAS* mutations (KRAS/MEK/ERK signaling pathway), AMPK signaling pathway, Wnt-β catenin pathway, *NFAT5, PDK1, LDHA*, and *P4HA1* alongside the epigenomic regulators UHRF1/SIRT4 axis, LSD1, and miR-124 are implicated in shifting the metabolic balance to an anaerobic glycolytic cellular phenotype that helps in improved malignant potential. The actions of the above factors are mediated by the sustenance of HIF-α under a hypoxic environment leading to the transcription of genes involved in enhancing tumor invasiveness and spread (44).

Hypoxia (1% O_2_) in dendritic cells (DC) further leads to the induction of indolamine 2,3-dioxygenase dependent on the adenosine A3 receptor as shown by Xiang Song et al. (45) Although a stabilized HIF-α inhibits IDO enzyme of the tryptophan metabolic pathway (46) in glioblastoma cells (47), the immunosuppressive Kynurenine, and its metabolites have an independent mechanistic role in the TME of tissues such as PDAC. This has been proven by a study conducted by Witkiewicz AK et al. (48) in 36 patients with PDAC and five pancreatic cancer cell lines. All samples revealed an increased expression of the IDO enzyme. Functionally active variants of the *IDO2* gene were also detected in patients. This corroborates the significant role of Kynurenine and its metabolites in immune modulation in the biology of pancreatic cancer. The kynurenine pathway and mitochondrial metabolism are related *via* superoxide generation and mitochondrial entry of one-carbon units into the TCA cycle *via* α-ketoadipate. Though hypoxia and superoxide ions were not found to induce IDO1 expression, when cell cultures were transferred from a monolayer to an AI 3D culture, it was found that the IDO1-dependent kynurenine pathway was dramatically switched on. Predictably, kynurenine efflux increased and the same could be abolished by an IDO1 inhibitor and a JAK1 inhibitor (23). Overall, the contribution of the IDO-Kynurenine-NAD^+^ pathway in immune modulation, cell proliferation, and metastatic promotion is likely undermined in cancer of the pancreas.

## Genomic and transcriptomic influences of IDO-Kyn pathway in PDAC

Pancreatic cancer, specifically PDAC has variants in key genes that aid in malignant transformation and metastasis. The most common genes carrying pathogenic mutations or variations are *KRAS, TP53, SMAD4*, and *CDKN2A.* Even though consistent across 50-90% of samples, we are yet to defeat the outcome using targeted therapy, except for renewed optimism with *KRAS* G12C inhibitors. Lesser common pathogenic variants within the DNA Damage Repair (DDR) pathway or in the *BRCA1* or *BRCA2* genes however respond to platinum-based chemotherapy and Poly (ADP-ribose) polymerase inhibitors (PARPi) as evidenced by the POLO trial (19). The extensive effect of *KRAS* mutations on the metabolome of PDAC has been discussed earlier (44). Furthermore, intact p53 was found to mitigate IDO1 expression in lung cancer thereby preventing metastasis (49) hence loss of function of *TP53* is likely to induce IDO1 expression. Overall, every omic variation inadvertently affects the tumor metabolome.

The novel genes altered in PDAC have specific functions to execute in the immunological milieu of the TME. Further, Cullis J et al. describe the *immunologistics* of *KRAS* variations in pancreatic cancer cell lines. Oncogenic *KRAS* signaling leads to activation of a plethora of downstream molecules such as TGF-β, GM-CSF, IL-10, IL-6, and the ELR^+^ CXC chemokines CXCL1, CXCL2, CXCL5, and CXCL8. These independently influence immunomodulation of the TME by not only influencing recruitment and differentiation of immune cells but also by inducing immune suppression and promoting tumor growth (50). *KRAS* mutations were also found to downregulate MHC Class I and inhibit immunostimulatory IL-18 to evade immune cell attacks in cancer cell lines (51). Wild-type *TP53* has been shown to increase T cell infiltration in PDAC whereas mutant *TP53* eliminates suppression on IL-6 and induces NF-κB and TNF-α signaling promoting metastasis. *TP53* knockout mice also showed T cell differentiation into T_H_17 cells that play a role in immune privilege as described earlier in this review. Further, p53 influences the PDAC microenvironment through a vast network of microRNAs, namely miR-34a, miR-21, miR-203, miR-128, miR-192, miR-200a, miR-200c, miR- miR-29. *MDM2* that suppresses action of *TP53* is in turn regulated by miR-29, miR-125a, miR-143, miR-145, and miR-365. miR-145 and miR-135 are specifically influence cellular glycolysis and TCA cycle to promote metastasis and growth of PDAC cells. Interestingly, gain of function (GOF) mutations in *TP53* inhibit p73 preventing binding of nuclear factor-Y transcription factor (NF-Y) which increases gene expression of PDGFR-β that is required for development of the fibrotic PDAC TME (52). *SMAD4* is a target of TGF-β and its loss is observed in more than half of PDAC cases. Principe DR et al. show that samples with a loss of *SMAD4* had lower T cell infiltrates irrespective of addition of neoadjuvant chemotherapy and cell culture from human pancreatic cancer cell lines showed a reduction in IFN-γ. *SMAD4* loss also impaired function of members of the CCL/CXCL chemokine family and Interleukin cytokine family. The role of *SMAD4* in modulating the immunogenicity of PDAC and pivoting the efficacy of immunotherapy is indisputable (53).

## Therapeutic potential of metabolome and clinical trials

Tryptophan and IDO-mediated immunosuppression within tumor cells and draining lymph nodes place IDO as a potential target to reverse suppression and augment immune-mediated anti-tumor interventions (54–56). Clinical trials have been investigating IDO1 inhibitors with positive and negative associations as results. Garber et al. (57) describe the enzyme and its activities in cancer as a “black box”. It is imperative that we have a better understanding of enzymology, molecular biology, and TME characteristics to employ successful IDO1 inhibitors in the clinic. The keyword ‘IDO1’ returns 47 clinical trial results from https://clinicaltrials.gov/.The list of active, recruiting, enrolling by invitation, and completed trials (n=25) are listed in **Table 1**.

**Table 1:** List of Clinical Trials investigating IDO inhibitors and NAMPT inhibitors.

DRUG	CLINICAL TRIAL ID	PHASE	STATUS	INDICATION	STUDY COHORT AND LOCATION
Epacadostat	NCT03516708	Phase I	Recruiting	Locally advanced rectal cancer	39 Participants, Washington University Patients only Location: United States
	NCT03322540	Phase II	Completed	Lung cancer	154 Participants Locations: Australia, Canada, Denmark, Estonia, Ireland, Israel, Italy, Japan, Korea, Republic of, Malaysia, Poland, Russian Federation, Spain, Switzerland, Turkey, Ukraine, United Kingdom, United States
Epacadostat+ Pembrolizumab	NCT03374488	Phase III	Completed	Urothelial cancer (UC)	84 Participants Location: Australia, Canada, Denmark, France, Germany, Hungary, Ireland, Israel, Italy, Japan, Korea, Republic of, Netherlands, Russian Federation, Spain, Taiwan, Turkey, United Kingdom, United States
	NCT02862457	Phase I	Completed	Advanced solid tumors	34 participants Location not Provided
	NCT03322566	Phase II	Completed	Lung cancer	233 Participants Location: Australia, Canada, Hungary, Ireland, Israel, Italy, Korea, Republic of, Mexico, Russian Federation, Spain, Taiwan, Turkey, United Kingdom, United States
	NCT03532295	Phase II	Recruiting	Recurrent gliomas	48 Participants Location: United States
	NCT03493945	Phase I/II	Recruiting	Solid tumor	113 Participants Location: United States
Epacadostat+ Pembrolizumab/ chemotherapy	NCT03328026	Phase I/II	Recruiting	Metastatic or locally recurrent breast cancer patients	36 Participants Locations: United States
	NCT03322384	Phase I/II	Completed	Advanced solid tumors lymphoma	20 Participants Locations: United States
Epacadostat+ Rapamycin	NCT03372239	Phase I	Completed	Drug safety trial	48 Participants Locations: Canada
Epacadostat+ INCMGA00012 + RT + bevacizumab	NCT03852446	Early phase I	Completed	Drug safety trial	56 Participants Locations: United States
Epacadostat+M7824 + BN-Brachyury + ALT-803 + Epacadostat (Immunotherapy)	NCT04049669	Phase II	Recruiting	Progressive brain tumors or newly diagnosed DIPG	140 Participants Locations: United States
Epacadostat+ INCMGA00012, Epacadostat 600 mg BID, SV-BR-1-GM combination	NCT02073123	Phase I/II	Completed	Advanced or metastatic melanoma	132 Participants Locations: United States
Epacadostate+ Intralesional SD101, Radiotherapy	NCT01560923	Phase II	Completed	Refractory metastatic prostate cancer	47 Participants Locations: United States
Indoximod	NCT03378310	Phase I	Completed	Drug safety trial	16 Participants Locations: United States
	NCT03374228	Phase I	Completed	Drug safety trial	7 Participants Locations: United Kingdom
	NCT03312426	Phase I	Completed	Drug safety trial	32 Participants Locations: United States
Indoximod+ Pembrolizumab/ nivolumab	NCT03362411	Phase I	Completed	Drug safety trial	40 Participants Locations: United States
Indoximod+ Temozolomide	NCT03247283	Phase I	Completed	Cancer	9 Participants Location: United States
Linorodostat (BMS-986205,ONO-7701)	NCT03792750	Phase I/ II	Completed	Advanced malignant solid tumors	12 Participants Locations: China
	NCT03459222	Phase I/ II	Recruiting	Advanced malignant tumors	225 Participants Locations: Australia, France, Italy, Spain, Switzerland, United Kingdom, United States
	NCT03519256	Phase II	Completed	BCG-unresponsive, high-risk, non-muscle invasive bladder cancer	69 Participant Locations: Argentina, Australia, Brazil, Canada, Chile, China, France, Hong Kong, Italy, Mexico, Netherlands, Russian Federation, Spain, Turkey, United Kingdom, United States
	NCT03346837	Phase I	Completed	Malignancies multiple	53 Participants Location: United States
	NCT03661320	Phase III	Recruiting	Muscle-invasive bladder cancer	861 Participants Locations: Argentina, Australia, Austria, Belgium, Brazil, Canada, Chile, Colombia, Finland, France, Germany, Greece, Israel, Italy, Japan, Korea, Republic of, Mexico, Netherlands, New Zealand, Norway, Portugal, Romania, Russian Federation, Singapore, Spain, Taiwan, United Kingdom, United States
Linorodostat (BMS-986205, ONO-7701)+Nivolumab	NCT04047706	Phase I	Recruiting	Glioblastoma	30 Participants Location: United States
	NCT03936374	Phase I	Completed	Drug safety trial	16 Participants Location: United States
	NCT02048709	Phase I	Completed	Recurrent advanced solid tumors	22 Participants Location: United States
Linorodostat (BMS-986205, ONO-7701)+Nivolumab/BCG	NCT02867007	Phase I	Completed	Locally advanced or metastatic solid tumors	36 Participants Locations: France, United States
Linorodostat (BMS-986205, ONO-7701)+Itraconazole/rifampin	NCT00435084	Phase III	Completed	B-Cell Chronic Lymphocytic Leukemia	10 Participants Location: United Kingdom
Linorodostat (BMS-986205, ONO-7701)+Nivolumab/chemotherapy	NCT00432107	Phase II	Completed	Melanoma	25 Participants Locations: Austria, France, Germany, Switzerland
Linorodostat (BMS-986205, ONO-7701)+Nivolumab/radiotherapy or chemoradiotherapy	NCT00431912	Phase II	Completed	Cutaneous T-Cell Lymphoma	25 Participants Locations: Austria, France, Germany, Switzerland
Linorodostat (BMS-986205, ONO-7701)+Omeprazole	NCT04914845	Phase I	Recruiting	Acute Myeloid Leukemia	40 Participants Location: United States
Navoximod (GDC-0919, NLG-919)	NCT04281420	Phase I	Recruiting	Solid Tumor	70 Participants Locations: China,Taiwan
KHK2455+ Avelumab	NCT03921879	Phase I	Recruiting	Lymphoma	50 Participants Location: United States
KHK2455+ Mogamulizumab	NCT02867007	Phase I	Completed	Locally advanced or metastatic solid tumors	36 Participants Locations: France, United States
APO866	NCT00435084	Phase III	Completed	B-Cell Chronic Lymphocytic Leukemia	10 Participants Location: United Kingdoms
	NCT00432107	Phase II	Completed	Melanoma	25 Participants Location: Austria, France, Germany, Switzerland
	NCT00431912	Phase II	Completed	Cutaneous T-Cell Lymphoma	25 Participants Location: Austria, France, Germany, Switzerland
KPT-9274	NCT04914845	Phase I	Recruiting	Acute Myeloid Leukemia	40 Participants Location: United States
ATG-019	NCT04281420	Phase I	Recruiting	Solid Tumor	70 Participant Locations: China, Taiwan
OT-82 Dose Escalation	NCT03921879	Phase I	Recruiting	Lymphoma	50 Participants Location: United States

Epacadostat is the most investigated IDO1 inhibitor in cancer. However, there is only one study on metastatic pancreatic cancer [Epacadostat, Pembrolizumab, and CRS-207, With or Without CY/GVAX Pancreas in Patients With Metastatic Pancreas Cancer (NCT03006302)]. As described in this review, IDO inhibitors may also have to be combined with serine and glycine restriction to block one-carbon unit generation and reversal of immune suppression. Inclusion of the molecular profile of tumor cells and variables influencing TME for example Immunoscore may help with a better-targeted selection of patients for Randomised Controlled Trials (RCTs) that are more likely to give results.

Owing to unimpressive Phase III trials of IDO1 inhibitors as single agents, Shao J et al. described a novel method wherein the inhibitor was loaded onto hyaluronic acid-modified nanomaterial graphene oxide (HA-GO) in conjunction with ongoing CAR-T cell therapy. The authors found that by inhibiting IDO so, CAR-T cells were more efficacious *in vivo* and *in vitro* (58). The safety and toxicity profile of combinatorial therapies are still being investigated, but the future is promising.

NAD^+^ being a crucial coenzyme cofactor generated by the kynurenine pathway, it is only right to investigate potential inhibitors. NAMPT regulates intracellular NAD concentration hence NAMPT inhibitors FK866/APO866, CHS-828, KPT-9274, and OT-82 are undergoing Phase I/II clinical trials (59–61).

## Conclusion

Pancreatic cancer is a poster child for unsuccessful targeted or immune checkpoint inhibitor therapeutic strategies. Javadrashid D et al. (62) highlight the challenges and have compiled the myriad factors influencing pancreatic cancer and the immune microenvironment. Efforts are to be made to improve survival outcomes by exploring novel treatment molecules and protocols, apart from standard of care. This review explores a novel avenue by highlighting the power of metabolomic influence, specifically the tryptophan metabolic cascade over the tumor immune microenvironment of PDAC. The immunosuppression offered by the downstream actions of IDO1 is credited to the activation of GCN2, inhibition of mTOR pathways which consequently steer tryptophan degradation, and Kynurenine pathway metabolites induced AhR activation (30). It is evident that the effector T cell repertoire is significantly restrained and incapacitated by Kynurenine and allied metabolites leading to immune suppression of the TME. IDO1 and NAMPT inhibitors as combinatorial therapy is postulated to be lucrative in the therapeutics of solid tumors. It is therefore clinically essential to expand the investigation of these agents into PDAC, a classic example of immune-transformed (63) and challenged cancer.

## Author contributions

All authors listed have made a direct and intellectual contribution to the work and approved it for publication.
